# Autobiographical memory loss following a right prefrontal lobe tumour resection: a case report and review of the literature

**DOI:** 10.1007/s00381-017-3380-7

**Published:** 2017-03-21

**Authors:** A. A. B. Jamjoom, P. Gallo, J. Kandasamy, J. Phillips, D. Sokol

**Affiliations:** Royal Hospital for Sick Children, 9 Sciennes Rd, Edinburgh, EH9 1LF UK

**Keywords:** Right prefrontal lobe, Memory loss, Astrocytoma, Episodic memory

## Abstract

**Introduction:**

The right prefrontal lobe has not traditionally been considered eloquent brain. Resection of tumours within this region does not typically lead to permanent functional impairment. In this report, we highlight the case of a patient who developed autobiographical memory loss following an uncomplicated resection of a right prefrontal tumour.

**Case material:**

A previously fit and well 15-year old presented with a persistent right-sided headache. An MRI demonstrated an expanded right mid-frontal gyrus with changes consistent with a low-grade tumour. The patient underwent a right-sided craniotomy and resection of the lesion which was confirmed as a WHO grade II diffuse astrocytoma. Postoperatively, the patient reported profound retrograde amnesia for a range of memory components, in particular autobiographical memory and semantic memory. Postoperative imaging showed a good resection margin with no evidence of underlying brain injury. Over an 18-month period, the patient showed no improvement in autobiographical memory; however, significant relearning of semantic knowledge took place and her academic performance was found to be in line with expectations for her age.

**Conclusion:**

In this report, we discuss a case and review the literature on the role of the right prefrontal cortex in memory and caution on the perception of right prefrontal non-eloquence.

## Case report

### History

**Fig. 1:**
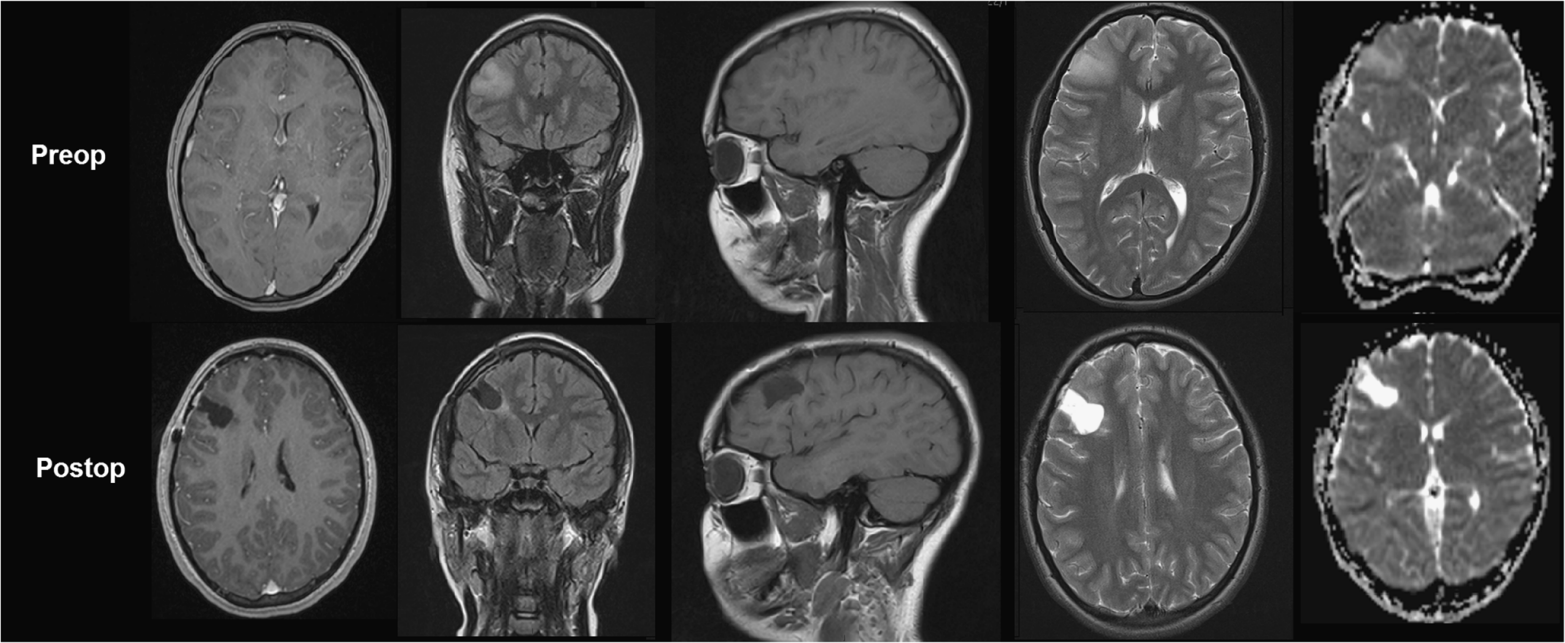
Preoperative and 3-month postoperative MRI images from VF including (*from left to right*) axial T1 post-gadolinium, coronal FLAIR MRI, sagittal T1, axial T2 and axial apparent diffusion coefficient (ADC) maps

A 15-year-old right-handed female (VF) presented in 2012 with a history of weekly headaches over the right side of her head and with a sudden onset of diminished auditory acuity in her right ear when she was wearing headphones. She perceived music in the right ear as a noise rather than music. There was no history of developmental, psychological or school difficulties. Audiological assessment found normal neurological responses to sound stimuli bilaterally suggesting normal hearing function (10 dB at 1 kHz and 20 dB at 4 kHz). A subsequent brain MRI showed the presence of an expanded right mid-frontal gyrus with changes extending from the cortex into the subcortical white matter consistent with a possible low-grade tumour (Fig. 1). Initially, VF was followed up with serial imaging until slight growth of the lesion was observed 14 months after diagnosis. She underwent a right-sided craniotomy and resection of the progressive right frontal lesion in February 2014. Histopathological analysis confirmed a WHO grade II diffuse astrocytoma.

Postoperatively, VF reported profound retrograde amnesia for a range of memory components, in particular, autobiographical memory and semantic memory. For example, she could not recall her parents, life history or friends. She could not recall her way around school and reported loss of knowledge such as her time table or how to make a cup of tea. Imaging demonstrated a good radiological resection of the lesion with no evidence of surrounding brain injury (Fig. 1). VF underwent no additional therapy following her surgery as follow-up imaging demonstrated a good resection and no tumour recurrence. Interestingly, her subjective hearing loss normalised after surgery.

### Neuropsychological assessment

VF underwent comprehensive neuropsychological assessment at a month, 6 months and 18 months after surgery. VF reported no improvement in autobiographical memory; however, significant relearning of semantic knowledge took place and VF’s academic performance was found to be in line with expectations for her age. VF reported ongoing difficulties in attention, prospective memory, poor organisational skills and emotional lability. VF’s last assessment indicated general intellectual abilities, executive skills and memory abilities in the average range, demonstrating age-appropriate skills in new learning and laying down new memories. Significant difficulties were identified in attention (particularly sustained, switching and dual-task attention).

## Discussion

VF’s case report demonstrates the dramatic disruption of episodic memory following the uncomplicated resection of a right-sided frontal lesion. Within neurosurgery, damage to the right prefrontal cortex is not commonly associated with significant memory impairment. However, a review of the literature demonstrated several studies and case reports looking at the subject of memory and the right frontal lobe. In 1996, Schacter described a case report of a patient who demonstrated striking patterns of false recognition following a right frontal lobe infarction [1]. The authors put the patient through a catalogue of cognitive tests and concluded that the patient’s high false recognition rate may be due to the loss of the right frontal lobe’s retrieval capacities which are vital for item recognition. A further lesion study by McDonald and colleagues looked to compare episodic memory in patients with left and right frontal lobe lesion resections for intractable epilepsy [2]. Interestingly, the patients with right frontal lobe lesions showed impairment of recognition memory compared to the other left frontal lobe and control groups.

In the last 15 years, there has been growing neuroimaging evidence on the role of the right prefrontal lobe in episodic memory. The activation of the prefrontal cortex in episodic memory retrieval has been a consistent finding [3]. However, teasing out the exact process and roles of the right prefrontal cortex remained. Over the convening years, the right prefrontal cortex has been demonstrated to be part of a network involved in retrieval of episodic memory, in particular, the monitoring of retrieved information [4, 5]. Studies have focused on elucidating episodic memory retrieval mode which refers to a cognitive state where an individual holds a past episode in focal attention and treats incoming information as retrieval cues [6]. A study by Henson using functional MRI found dissociation in activity between the dorsal and ventral regions of the prefrontal cortex with the dorsal region associated with increased contextual monitoring and the ventral region being independent of task instructions [7]. Similarly, a positron emission tomography study by LePage identified three regions within the right prefrontal cortex in playing a role in episodic memory retrieval: frontal pole [Brodmann’s area (BA) 10], frontal operculum (BA 47/45) and lateral dorsal area (BA 8/9) [6]. This process has been found to be suppressed in patients with post-traumatic stress disorder (PTSD), who have impaired memory, with deactivation of the right dorsolateral prefrontal cortex during memory retrieval compared to controls [8]. Similarly, patients with right frontal lobe tumour resections had impaired recollection-based verbal memory compared to healthy controls [9].

Interestingly, Ragilo and colleagues found activation of the right middle frontal gyrus and, in particular, the right precentral gyrus in volunteers undergoing active music therapy [10]. A study by Janata looked at the association between autobiographical memory and music and concluded that the medial prefrontal cortex may play a role [11]. VF had presented with reduced auditory acuity in her right ear which may have been related to the tumour. However, this subjective auditory impairment improved after surgery while VF’s episodic memory impairment remained problematic which points away from a direct anatomical correlation.

The evidence for the role of the right prefrontal cortex in episodic memory retrieval may indicate the potential biological cause for VF’s memory impairment. However, the exact cause of the severity of VF’s deficit is to be explained. This is not fully accounted for by her performance on standardised neuropsychological tests. Similarly, we were unable to undertake functional MRI on VF. This investigation could have helped provide more information on function localisation. However, given VF’s preoperative function, the anatomical location of the lesion and planned surgical approach, functional imaging is not routinely undertaken in our hospital. While we cannot entirely rule out a biological explanation, psychogenic or functional mechanisms should be considered. VF’s day-to-day functioning is mostly affected by poor attention which may impact on new learning as well as reported difficulties in executive functioning and psychological status [12]. However, based on VF’s neuropsychological assessment, associated psychological factors may also contribute to reported difficulties in executive function. This is likely to be something of a vicious cycle with psychological factors exacerbating underlying vulnerabilities in attention.

The right prefrontal cortex has long been considered by neurosurgeons to be a non-eloquent and relatively safe for resection. However, the prefrontal cortex is essential for attention control, manipulation of stored knowledge and modulation of complex actions, cognition, emotion and behaviour. This case study demonstrates a potential risk which should be taken into account in preoperative planning and counselling.
